# Accelerated Wound Healing of Tetrahedral-Framework Nucleic Acid Nanozymes with High Penetration and Antioxidant Capacity

**DOI:** 10.3390/nano14211693

**Published:** 2024-10-23

**Authors:** Shiyu Lin, Qian Liu, Yu Xie, Qi Zhang

**Affiliations:** 1 National Center for Stomatology, National Clinical Research Center for Oral Diseases, Shanghai Key Laboratory of Stomatology, Department of Oral Surgery, Shanghai Ninth People’s Hospital, Shanghai Jiao Tong University School of Medicine, College of Stomatology, Shanghai Jiao Tong University, Shanghai 200011, China; 122015@sh9hospital.org.cn; 2 Department of Stomatology, Tongji Hospital, School of Medicine, Tongji University, Shanghai 200070, China; fmmuliuqian@foxmail.com; 3 Department of Oral and Maxillofacial Implantology, Shanghai PerioImplant Innovation Center, Shanghai Ninth People’s Hospital, Shanghai Jiao Tong University School of Medicine, College of Stomatology, Shanghai Jiao Tong University, National Center for Stomatology, National Clinical Research Center for Oral Diseases, Shanghai Key Laboratory of Stomatology, Shanghai Research Institute of Stomatology, Shanghai 200011, China; xieyu1997@sjtu.edu.cn

**Keywords:** framework nucleic acids, burn wound, oxidative imbalance, DNA nanozymes

## Abstract

The wound repair process usually leads to a non-functioning mass of fibrotic tissue because of the oxidative imbalance of deep tissue layers. However, how to improve the penetration of active ingredients into deeper layers and regulate oxidative imbalances to create a regenerative microenvironment still remains a challenge. In this study, we designed a novel tetrahedral-framework nucleic acid (tFNA) nanozyme that could penetrate the skin/mucosa barrier as deep as 450 μm within 24 h. We also demonstrated the protective role of tFNAs on the mitochondrial structural and functional integrity and inhibition of reactive oxygen species production to repair oxidative imbalances through ERK1/2-Nrf2-HO-1 during repair processes. It was found that the proliferative state and the migration ability of postburn cells in vitro were accelerated, and the early closure of wounds in vivo was significantly promoted. This study therefore provides a promising strategy to efficiently regulate the oxidative imbalances in the deep layers of the skin during wound healing.

## 1. Introduction

Burns are usually skin/mucosal wounds caused by heat, electricity, chemicals, and radiation [1]. A defect in the skin/mucosal barrier leads to the exposure of deep tissues and a decrease in local immunity, which is accompanied by an inflammatory response, laying the groundwork for subsequent localized or even systemic infections [2,3]. During burn injury, the conduction of temperature, diffusion of chemicals, and penetration of radiation all lead to cellular dysfunction and oxidant microenvironment disorder in tissues in the deeper part of the wound. Therefore, how to efficiently penetrate into deep tissues and mobilize the regenerative state is crucial for the healing of burn wounds.

Wound dressings (such as collagen, chitosan, alginate, and cellulose) are the most common method for protecting wounds and providing a suitable environment. The design of commonly used biomaterials mainly focuses on coagulation, local immune microenvironment, local cell proliferation and migration, and the promotion of vascular formation in burn wounds [4,5]. However, the insufficient efficiency of materials and drugs in tissue infiltration and intracellular delivery limits their ability to regulate the microenvironment and activate cell activity in deeper tissue cells. Compared with conventional materials, nanomaterials exhibit marked effects in promoting wound closure, eliminating local oxidative stressors, and inhibiting infections by adjusting the drug morphology, enhancing the concentration of effective drugs, precisely controlling the release rate, or targeting specific locations [6]. However, deep tissue restoration of burn wounds requires nanomaterials to penetrate the skin or mucous membranes more efficiently. The penetration of nanomaterials is influenced by their particle size, properties, and solubility, with smaller particles and those containing soluble substances and metals penetrating the skin more easily. Nucleic acid-relevant nanomaterial is one of these desirable and ideal materials. Directing the recognition and self-assembly of DNA molecules based on their inherently embedded complementary pairing leads to precise polymorphic nanoscale constructions. For example, star nucleic acids composed of gold NPs and oligonucleotides can penetrate the cuticle and epidermal barriers on their own [7]. Studies revealed better drug accumulation of doxorubicin-loaded framework nucleic acid (FNA) in mouse melanoma models than liposomes and polymer nanoparticles [8].

A tetrahedral-framework nucleic acid (tFNA), as a simple and classical three-dimensional structure, exhibits the characteristics of editable precision size, easy modification of sites, and high synthetic stability [9]. Its sharp-corner structure is more conducive to its proximity to penetration through the cell membrane for the efficient delivery of functionalized molecules or drugs assembled on it without the assistance of transfection reagents [10]. By changing the framework nucleic acid morphology, modifying the motifs and aptamers, and regulating the ion concentration and species of the solution, framework nucleic acids have more possibilities for biological applications [11,12]. The tetrahedral-shaped framework nucleic acid itself, due to its deoxyribonucleotide structure, contains groups reacting with reactive oxygen molecules. Reaction with reactive oxygen species and its high cell-entry efficiency give it the potential to reduce reactive oxygen species production in the cytoplasm. In cells with burn wounds, their mitochondrial integrity is impaired, which triggers excessive reactive oxygen species production. Therefore, our work envisages whether the properties of tFNAs can ameliorate this situation [13,14].

In this work, we took the transdermal ability of a tetrahedral-frame nucleic acid (tFNA) as the entry point to verify its antioxidant role in regulating oxidative balance after burn. After successful synthesis and characterization of the tFNA, we adopted skin penetration assays, burn model establishment, mitochondrial integrity detection, ROS examination, and in vivo wound healing experiments to investigate the protective role of framework nucleic acids on burn wound healing at the material–cell–molecule–animal levels.

## 2. Materials and Methods

### 2.1. Materials

The following materials were used: DNA single strands (Sangon Biotechnology, Shanghai, China); RT-PCR primers (Sangon Biotechnology); Nrf2 (1:1000; #12721; Cell Signaling Technology, Danvers, MA, USA); HO-1 antibody (1:1000; #82551; Cell Signaling Technology); ERK1/2 inhibitor (5 nM; LY3214996; Selleck Chemicals, Houston, TX, USA); PCNA antibody (1:500; ab92552; Abcam, Cambridge, UK); cell cycle detection kit (Beyotime Biotechnology, Nantong, China); ROS detection kit (Solarbio Life Science, Beijing, China); and mitochondrial membrane potential/apoptosis detection kit (Beyotime Biotechnology).

### 2.2. Synthesis of Tetrahedral-Framework Nucleic Acids

In the process of synthesizing tetrahedral-framework nucleic acids, four specially designed DNA single strands (Table 1) were first required to be solubilized and quantified before being added to the buffer in equimolar proportions. After gently shaking, mixing, and centrifugation, the mixture of single strands was accomplished by uniform heating at 95 °C for 10 min, followed by uniform cooling at 4 °C for 20 min. Finally, the synthesized tetrahedral-framework nucleic acids were filtered.

### 2.3. Characterization of Tetrahedral-Framework Nucleic Acids

We used high-performance capillary electrophoresis (HPCE) to electrophoretically separate and detect nanoscale DNA single strands and DNA framework nucleic acids by using a capillary as a separation channel under a high-voltage electric field, utilizing differences in the nature of the charge and the size of the particles. PAGE gels were prepared using TAE as the base liquid, and s1, s2, s3, s4, and tetrahedral-framework nucleic acid samples were uploaded with 5 µL of nucleic acid samples each time in conjunction with 1 µL of 6× loading buffer. Vertical electrophoresis was performed for 90 min in TAE electrophoresis solution at a constant voltage of 90 V and stained with Gel-red, followed by exposure. The positions of the DNA single strands and framework nucleic acid bands were as expected. The particle size of the tetrahedral-framework nucleic acids was characterized by dynamic light scattering, their potential by zeta potential testing, and their morphology by transmission electron microscopy.

### 2.4. Cell Culture and Injury Model

L929 cells were used as model cells. The experimental methods included a blank control group, an injury group, and a post-injury tFNA treatment group. We first used 50 °C, 60 °C, and 65 °C to explore suitable temperatures, and then, combining the results, we chose 60 °C as the subsequent injury condition. The injury group was treated with 60 °C medium for 1 min to simulate a chemical burn scenario, whereas the post-injury tFNA treatment group was treated with 60 °C medium for 1 min, and then the cells continued to be cultured in medium containing 250 nM tFNA. During the inhibition of ERK1/2, we used 5 μM concentration for 24 h of pretreatment.

### 2.5. Cell Uptake

L929 cells were starved of 0% medium for 24 h before being cultured in medium containing 250 nM Cy5-modified tFNA for 48 h. Afterwards, they were washed and fixed with 4% paraformaldehyde, their cytoskeleton was stained with FITC phalloidin, the nuclei were stained with DAPI, and the cells were visualized by laser confocal microscopy.

### 2.6. Immunofluorescence Staining of Nrf2 and HO-1

Cells were fixed using paraformaldehyde for 30 min after completion of cell processing. Next, wells were punched using 0.5% Triton X-100 at room temperature and closed using goat serum at the same temperature for 30 min. The primary antibody (Nrf2, HO-1) was incubated for at least 12 h at 4 °C, and then the red fluorescent IgG secondary antibody was added and incubated. After that, the cells were stained using phalloidine and DAPI for 30 min and 5 min, respectively, at room temperature, protected from light, and finally observed under a laser confocal microscope.

### 2.7. CCK8 Analysis

Cells were inoculated into 96-well plates at 5 × 10^3^ cells/well. After 24 h, the medium was removed. Next, 100 μL per well of fresh serum-free medium with 10 μL of CCK8 reagent was added. We incubated at 37 °C and 5% CO_2_ for 1 to 4 h. Absorbance was measured at 450 nm using an enzyme meter. According to the number of cells in each well and the measured OD value, the “cell number–OD value” curve can be plotted, and the corresponding cell viability can be derived.

### 2.8. Flow Cycle Assay

After trypsin digestion of the cells, centrifugation was performed, and the cells were resuspended. They were washed using PBS and then fixed with 70% ethanol. The next day, cells were centrifuged, washed again, and incubated with 0.5 mL of propidium iodide staining solution. The cells were warm-bathed at 37 °C for 30 min protected from light, and then the red fluorescence was detected at 488 nm excitation wavelength using a flow cytometer while observing the light scattering.

### 2.9. Mitochondrial Membrane Potential Staining

Centrifugation at 1000× *g* for 5 min was performed on 24-well plates at the end of 48 h of treatment. The plates were stained with 188 µL Annexin V-FITC conjugate, 2 µL Mito-Tracker Red CMXRos stain, and 5 µL Hoechst stain in that order, gently mixed, and then incubated at room temperature and protected from light for 20–30 min; finally, they were observed under a fluorescence microscope.

### 2.10. Reactive Oxygen Species Detection

We tested the level of intracellular ROS through the Reactive Oxygen Species detection kit (Beyotime, China). Briefly, the medium was discarded after cell treatment, and then the cells were washed with PBS three times. Subsequently, we added DCFH-DA at a concentration of 10 μmol/L. After incubation at 37 °C for 20 min, the cells were washed three times with serum-free cell medium. The samples were observed and photographed using a fluorescence microscope. The excitation wavelength was 488 nm, and the emission wavelength was 525 nm.

### 2.11. Quantitative Real-Time PCR

FreeZol Reagent was used to purify RNA after collection of cells, following the instructions of the purification kit. After reverse transcription, samples were then mixed with primers, SYBR qPCR Master Mix, and enzyme-free water in specific ratios and subjected to real-time quantitative fluorescent PCR according to the Roche Instruments program to analyze gene expression. The primer sequences are shown in Table 2.

### 2.12. Scratch Experiment

Using the scratch migration healing assay, when the adult fibroblasts spread about 90% of the bottom area of the 12-well plate, starvation was carried out and uniform scratches were created, and then the corresponding treatment was given. The samples were captured several times during the 72 h of the experiment. The healing area of the scratch trauma was analyzed in detail using Image J software (V1.8.0).

### 2.13. ERK1/2 Inhibition

To explore the role of ERK1/2 in the regulation of mitochondrial membrane potential by tFNA, we treated the cells with an ERK inhibitor (5 nM working concentration) for 24 h after modeling and then added tFNA for 24 h to observe the changes in mitochondrial membrane potential under a fluorescence microscope.

### 2.14. In Vivo Penetration, Chemical Burn Modeling, and Wound Healing

The animal experiments for this research were approved by the Ethics Committee. To verify the penetration ability of tFNA, Cy5-labeled ssDNA and tFNA, both at a concentration of 1 μM, were incubated with mucosa for 24 h at 37 °C. The sample was embedded and sliced, and then the nuclei were stained with DAPI for 15 min before sealing. The sections were scanned by fluorescence microscopy and the depth of tFNA penetration into the mucosa was analyzed and quantitatively measured.

For the experiments, 8-week-old Sprague-Dawley rats (8 weeks, male) were randomly divided into two groups (*n* = 4). The rats were fed adaptively for 1 week before the oral burn model establishment. The oral ulcer model was performed in a sterile environment. After inhalation anesthesia with 5% chloral hydrate anesthesia, the rats were fixed in supine position, and the buccal mucosa was disinfected bilaterally by gently pulling the upper and lower jaws. Subsequently, the buccal mucosa was gently swabbed with a sterile cotton ball to keep it dry. Then, 3 mm large NaOH crystals were placed on the mucosa and held for 30 s before being removed. Subsequently, the wound was gently cleaned with saline. Next, 3–4 injection sites were selected under the mucosa at the wound edges, and 100 µL of framework nucleic acid (250 nM) was injected in a homogeneous manner, followed by slow withdrawal of the needle. Finally, an equal amount of 100 µL saline was injected into the contralateral buccal mucosa. In in vivo experiments, we collected standardizing photographs on day 1, day 3, day 5, day 7, and day 10. Adjusting the contrast and threshold to display the contour of the wound, the software can directly measure the pixel area after surrounding the wound contour (Image J, USA). The degree of wound healing is demonstrated by calculating the ratio of the wound area to the initial wound area. After collecting the samples, we embedded and sectioned them and performed HE staining, Masson staining, and immunohistochemical staining for PCNA.

### 2.15. Immunohistochemical Staining for PCNA

Samples were dewaxed and treated with gradient ethanol for antigen repair, blocked, and incubated with PCNA primary antibody at 4 °C for 12 h. After rewarming, the secondary antibody was incubated at room temperature for 30 min. After drying, the samples were blocked and scanned.

### 2.16. Statistical Analysis

All manipulations were repeated three or more times, and data results were expressed as mean ± standard deviation. One-way ANOVA was adopted for analysis, and statistically significant differences were considered to exist when the *p* value was less than 0.05. Data were collated and analyzed using SPSS 21.0 software, which was provided by SPSS Inc. (Chicago, IL, USA).

## 3. Results and Discussion

### 3.1. Synthesis and Characterization of Tetrahedral-Framework Nucleic Acid Nanozymes

**Figure 1:**
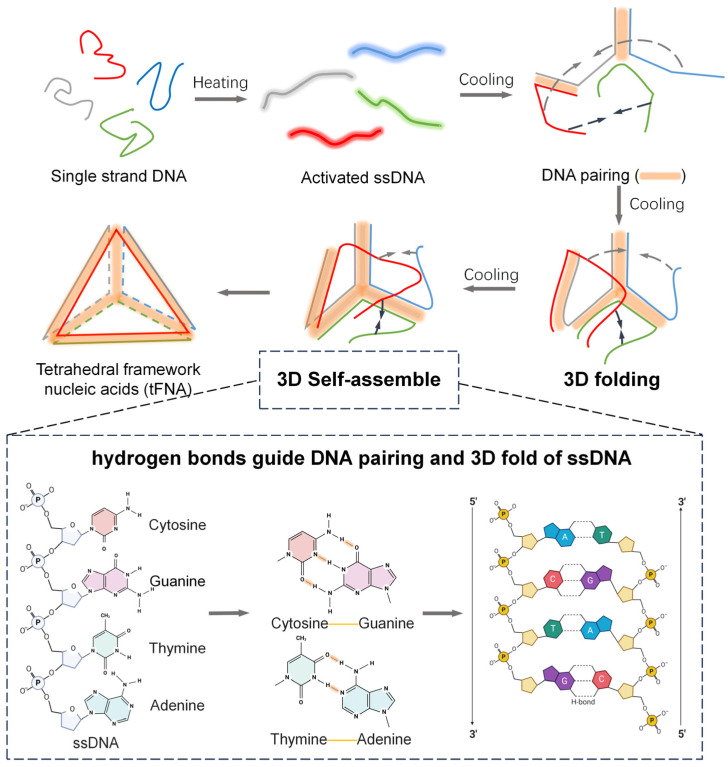
Schematic illustration showing the three-dimensional folding and self-assembly of specific DNA single strands to form tetrahedral-framework nucleic acids (tFNAs). DNA single-strands, which are stretched by heat, form complementary pairing with each other based on specific sequences during the cooling process, and the pairing with multiple single strands guides the single strands in three-dimensional folding and finally forms a simple and stable tetrahedral-framework nucleic acid structure.

The schematic diagram of the preparation of tetrahedral-framework nucleic acids is shown in Figure 1. DNA single strands opened at high temperature, and complementary sequences in the four single strands formed hydrogen bonds with each other during the cooling process. Three DNA single strands formed a preliminary structure at the two-dimensional level, and the fourth single strand guided it to fold towards the center, thus forming a three-dimensional structure.

**Figure 2:**
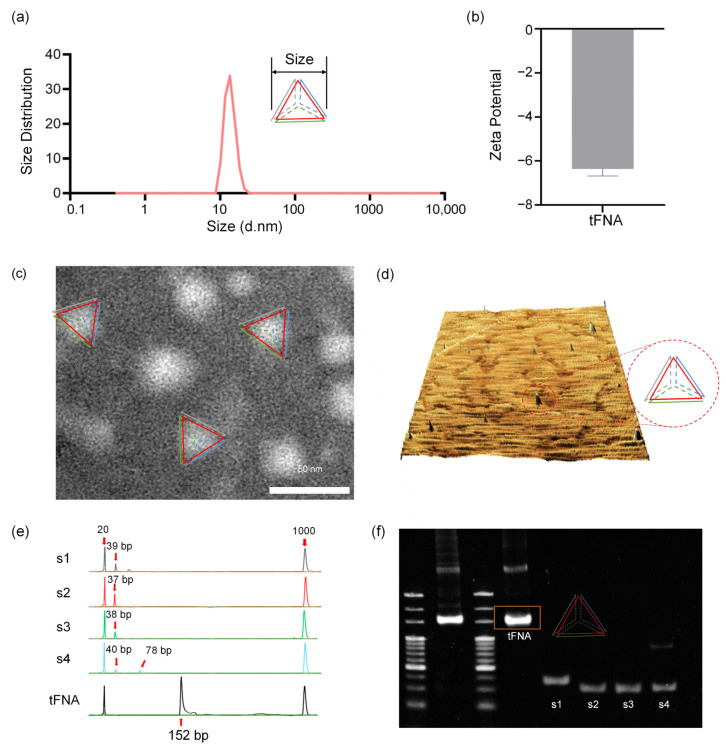
Assays verified the successful synthesis of tFNA. (**a**) Particle size detection of tFNA. (**b**) Zeta potential of tFNA. (**c**) TEM images visualizing the morphology and size of tFNA. (**d**) AFM examination for the morphology of tFNA. (**e**,**f**) HPCE and PAGE gel electrophoresis to separate and analyze DNA single strands and tFNA.

The successful synthesis of the framework nucleic acid was verified by the assays shown in Figure 1. Particle size and potential studies (Figure 2a,b) confirmed that tetrahedra are negatively charged nanomaterials with a particle size of approximately 20 nm. Transmission electron microscopy (Figure 2c), on the other hand, was used to visualize its morphology as approximately 20 nm tetrahedra. The graphic reconstruction of atomic force microscopy (Figure 2d) also preliminarily revealed the angular morphology of the DNA nanozymes. We adopted high-performance capillary electrophoresis and PAGE gel electrophoresis to separate and analyze the DNA single strands and tetrahedral-framework nucleic acids. The electrophoretic position and molecular weight in Figure 2e,f confirmed that all four single strands were involved in the synthesis of tetrahedral-framework nucleic acids (152 bp) and provided a basis for the three-dimensional folding of nucleic acid structures.

### 3.2. High Penetration of tFNA

**Figure 3:**
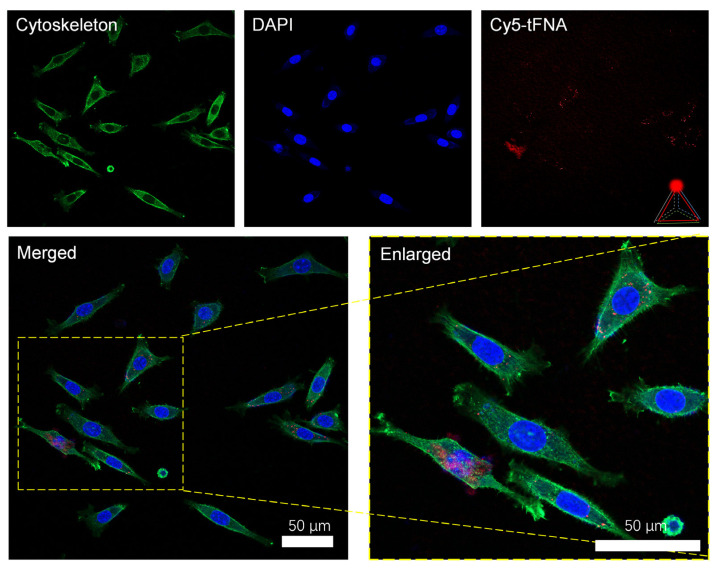
Efficient cellular uptake of tFNAs labeled by Cy5. Fluorescent tFNAs entered the cell in large quantities and were evenly distributed in the cytoplasm.

The Cy5 fluorescence-modified framework nucleic acids demonstrated better cell-entry efficiency and tissue penetration ability without requiring transfection agents. Ordinary nucleic acid materials cannot easily penetrate the cell membrane, but after folding into a tetrahedral-framework nucleic acid in three dimensions, we observed a large amount of fluorescent nucleic acids penetrating the cell membrane and dispersing in the cytoplasm (Figure 3).

**Figure 4:**
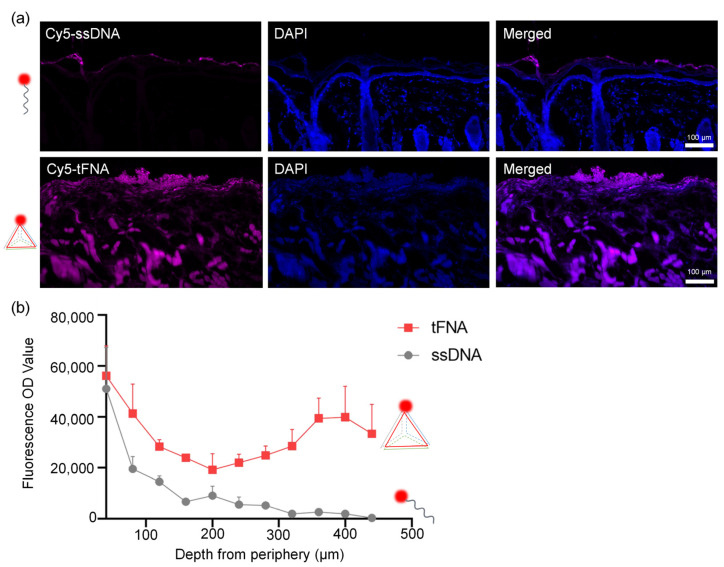
High penetration ability of tFNAs. (**a**) Depth of penetration of Cy5-ssDNA and Cy5-tFNAs into the mucosa. (**b**) Corresponding fluorescence semiquantitative measurement. Cy5-tFNAs could penetrate the skin as deep as 450 μm from the periphery within 24 h.

Figure 4 reveals the high penetration of tFNA nanozymes into the skin barrier. Cy5-labeled tFNAs could penetrate the skin as deep as 450 μm from the periphery within 24 h, while Cy5-labeled DNA single strands mainly stayed around the stratum corneum.

During burn injury, the conduction of temperature, diffusion of chemicals, and penetration of radiation all lead to cellular dysfunction and microenvironmental disorder in tissues (without obvious physical damage) in the deeper part of the wound [15,16]. Therefore, the penetration of drugs is crucial for deeper tissue. The penetration ability of nanomaterials is closely related to their size, morphology, charge, and composition. The tFNA constructed in this study has good cell membrane and skin barrier penetration ability with its tetrahedral edges and a size of about 20 nm. It could penetrate deeper tissues below the wound (such as blood vessels, collagen, glands, muscles, etc.) to achieve further effects on deeper injured tissues. The ability to enter fibroblasts without requiring transfection agents also allows tFNAs to function in the cytoplasm.

### 3.3. Framework Nucleic Acids Protect Mitochondrial Integrity While Reducing Reactive Oxygen Species Production

**Figure 5:**
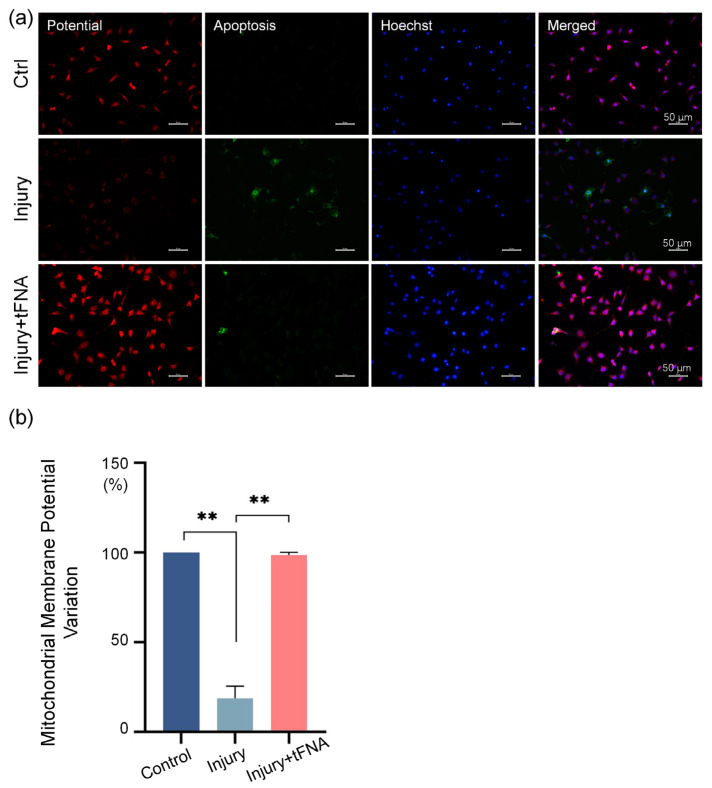
tFNA protects mitochondrial integrity. (**a**) Mitochondrial membrane potential in each group. (**b**) Semi-analysis of mitochondrial membrane potential. Mitochondrial membrane potential decreased when damaged, while tFNA increased mitochondrial membrane potential. (** *p* < 0.01).

Reactive oxygen species and mitochondrial membrane potential were examined to further explore the effect of tFNAs on the intracellular environment after burn injury. As shown in Figure 5a,b, the mitochondrial membrane potential in the cytoplasm decreased to 0.19-fold when damaged. tFNA ingestion increased the mitochondrial membrane potential of the cells to 0.99-fold.

**Figure 6:**
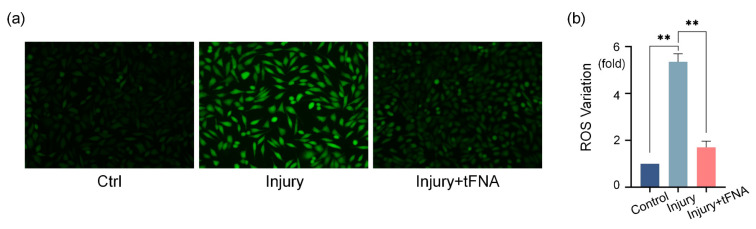
tFNA reduces reactive oxygen species production. (**a**) Reactive oxygen species production in each group. (**b**) Mean fluorescence intensity semi-analysis of reactive oxygen species production. Fluorescence intensity decreased in tFNA group when compared to damaged cell group. (** *p* < 0.01).

Along with mitochondrial damage, the concentration of reactive oxygen species (Figure 6a,b) in the cytoplasm increased, while tFNA entry into the cells effectively alleviated the production of reactive oxygen species. The fluorescence intensity decreased from 2.76-fold (MFI: 4952 ± 285) in the damaged cell group (relative to the control) to 2.13-fold (MFI: 3822 ± 143) in the tFNA group (relative to the control). We found that framework nucleic acids could effectively protect the mitochondrial membrane potential and reduce the production of reactive oxygen species.

Disruption of the oxidative–antioxidant balance occurs locally in burn wounds. The mitochondria are where reactive oxygen species are generated, and the stabilization of mitochondrial function maintains the oxidation–antioxidant balance and slows the formation of reactive oxygen species [17]. After cells are damaged by burn injury, the mitochondrial structure and function are damaged. Excessive ROS induce the opening of MPTP pores in the mitochondrial membrane, decreasing the membrane potential and further generating and releasing ROS [18,19]. Fusion and division processes promote the spread of mitochondrial damage, and the oxidative–antioxidant balance is completely disrupted, resulting in the disruption of the ratio of ROS to SOD, cytochrome C, and catalase, leading to cellular damage and apoptosis [20,21]. The reduction in reactive oxygen species production and the increase in antioxidant capacity after treatment with framework nucleic acids were verified.

### 3.4. Protein and Gene Changes Related to Reactive Oxygen Species Regulation

**Figure 7:**
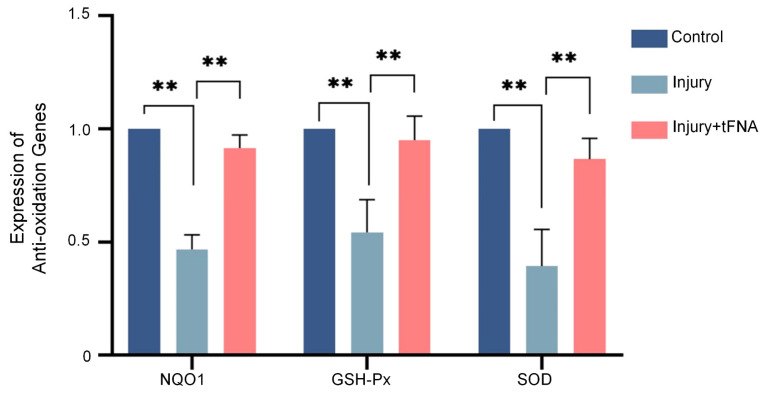
tFNA increased expression of anti-oxidation-related genes. Expression of *NQO1*, *GSH-Px*, and *SOD* genes significantly decreased after injury, and tFNA treatment reversed the trend. (** *p* < 0.01).

**Figure 8:**
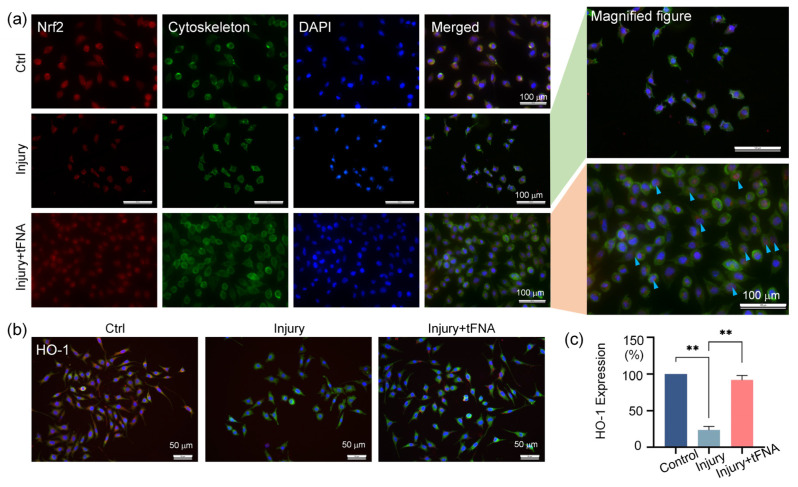
tFNA regulates anti-oxidation via Nrf2/HO1. (**a**) tFNA facilitated nuclear translocation of activated Nrf2. (**b**,**c**) Expression and semiquantitative analysis of HO-1 after burn injury and tFNA treatment. HO1 expression was attenuated after burn injury and up-regulated by tFNA. (** *p* < 0.01).

We further investigated the variation in Nrf2, HO1, and the expression of the *NQO1*, *GSH-Px*, and *SOD* genes related to anti-oxidation at the molecular level. The results are shown in Figure 7 and Figure 8. The expression of the *NQO1*, *GSH-Px*, and *SOD* genes significantly decreased after injury (by 0.69-fold, 0.34-fold, and 0.37-fold, respectively), and the tFNA treatment mitigated this decrease (up to 0.93-fold, 1.41-fold, and 0.94-fold, respectively).

As for Nrf2, the tFNA induced more Nrf2 for nuclear translocation (Figure 8a). Figure 8b,c revealed that HO1 expression was attenuated to 0.23-fold of that in the control group after burn injury and up-regulated to 0.92-fold by tFNA.

The expression of Nrf2 and HO-1 was activated after the addition of framework nucleic acids. Nrf2 and HO-1 are signaling systems for the cell when it responds to oxidative damage [22,23]. The antioxidant capacity of mitochondria, the main organelles involved in reactive oxygen species formation, is impaired when cells undergo increased oxidative stress. In response to oxidative damage, different MAPKs influence the development of oxidative damage through the activation and expression of Nrf2/HO-1 [24,25]. ERK activation ameliorates tissue damage and promotes the expression of lower Nrf2. Enhanced expression of Nrf2 is accompanied by nuclear translocation, which, when combined with antioxidant response elements, activates heme oxygenase (HO-1), NAD(P)H dehydrogenase, quinone 1 (NQO1), superoxide dismutase (SOD), and glutathione [26,27]. SOD and glutathione peroxidase (GSH-Px) are involved in ROS catabolism and scavenging [28,29].

**Figure 9:**
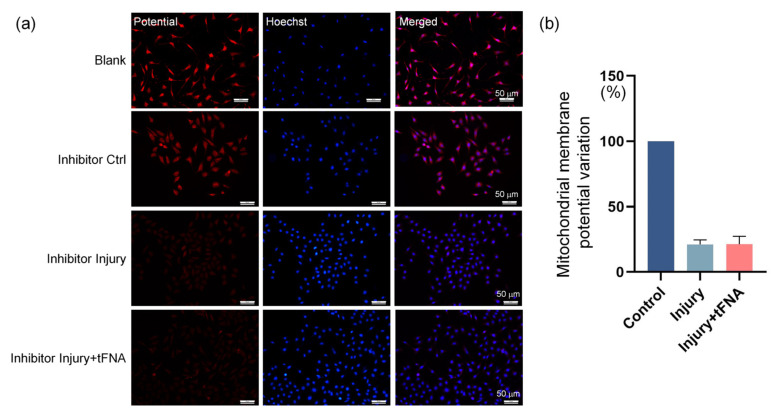
Mitochondrial membrane potential variation after ERK1/2 inhibition. (**a**) Treatment with tFNA in the presence of ERK1/2 inhibition did not significantly change the mitochondrial membrane potential. (**b**) Corresponding fluorescence semiquantitative measurement. Data are presented as mean ± standard deviation (SD) (*n* = 4).

The literature suggests that the ERK1/2-Nrf2 signaling pathway is closely related to cell proliferation, apoptosis, and oxidative damage. In the present work (Figure 9), the role of ERK1/2 in the tFNA mitigation of burn damage was validated. Upon inhibition of ERK1/2 by the inhibitor, we found a decrease in the cytoplasmic intracellular mitochondrial membrane potential. In contrast, treatment with tFNA in the presence of ERK1/2 inhibition did not significantly change the mitochondrial membrane potential. It can be speculated that, in addition to the antioxidant properties of framework nucleic acids directly reacting with reactive oxygen species, they also mobilize the cell’s own antioxidant system through the ERK1/2 signaling pathway [30,31].

### 3.5. Framework Nucleic Acids Reverse Damage of Burns to Cell Proliferation and Migration State

**Figure 10:**
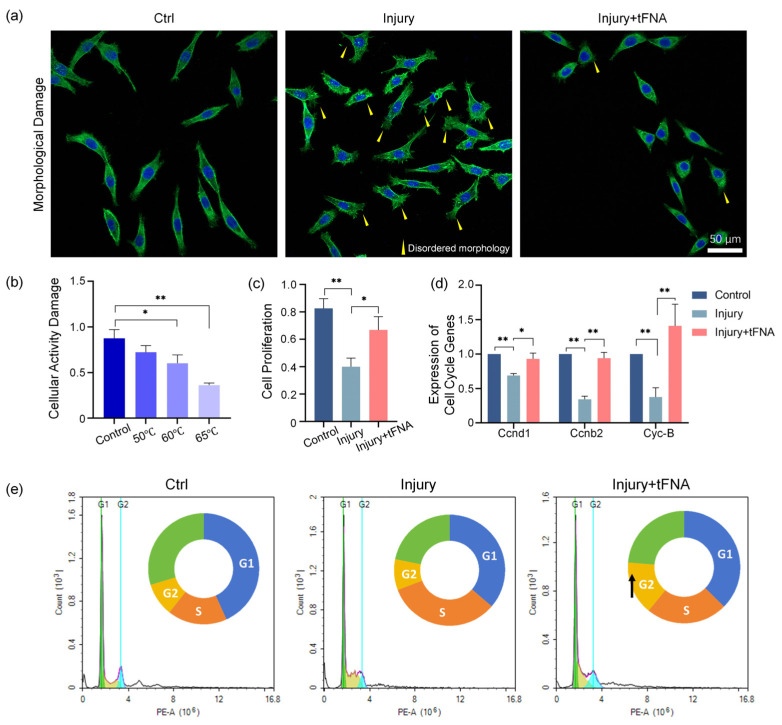
tFNA improves the activity and proliferation of injured cells. (**a**) Disordered cell morphology after preparation of an in vitro burn model. The yellow arrow points to disordered morphology. (**b**) Temperature gradient damage effect of burn model. (**c**) Improvement in burn cell activity and proliferative capacity by tFNA. (**d**) Variations in expression of cell cycle-related genes (*CCND1*, *CCND2*, *Cyc-B*). (**e**) Effect of tFNA on burn cell cycles. Wound healing closure assay and semiquantitative analysis after tFNA treatment. The black arrow indicates an increase in the proportion of G2 phase. Data are presented as mean ± standard deviation (SD) (*n* = 4). Statistical analysis: * *p* < 0.05; ** *p* < 0.01.

We induced short-duration damage to the fibroblasts at high temperatures to stimulate burns. The cells were heated at 50 °C, 60 °C, and 65 °C for 1 min, and CCK-8 was determined to assess the damage. As shown in Figure 10b, the activity of fibroblasts (CCK-8 results) decreased to 0.72 ± 0.07, 0.60 ± 0.08, and 0.36 ± 0.02 with increasing temperature compared to 0.87 ± 0.09 at 37 °C. As shown in Figure 10a, the cell spreading morphology and the cytoskeleton arrangement were affected after the cells underwent burn injury. The above results confirmed the successful establishment of the model. The cells in the tFNA treatment group exhibited normal cell morphology and an improved cytoskeleton to some extent. Under the action of tFNA, the shape and structure of the cells can be reversed and restored to a certain extent, and combined with the results of CCK-8 proliferation (Figure 10c), we can see the positive effect of tFNA on the survival of cells under thermal injury (CCK-8 value from 0.40-fold to 0.67-fold).

We further investigated the expression of genes related to proliferation. In Figure 10d, the expression of the *Cyclin D1*, *Cyclin D2*, and *Cyclin B2* genes significantly decreased after injury (to 0.69-fold, 0.34-fold, and 0.37-fold, respectively), and tetrahedron treatment mitigated this decrease (up to 0.93-fold, 0.94-fold, and 1.41-fold, respectively). Based on the results of cell proliferation, we analyzed the specific staging changes in cell proliferation during damage and tFNA treatment using flow cytometry (Figure 10e). The effect of tFNA shifted the damaged fibroblasts from a reduced apoptotic state to a proliferative state. The proportion of cells in the S and G2 phases increased from 32.90% and 9.00% to 23.42% and 15.47%, respectively.

**Figure 11:**
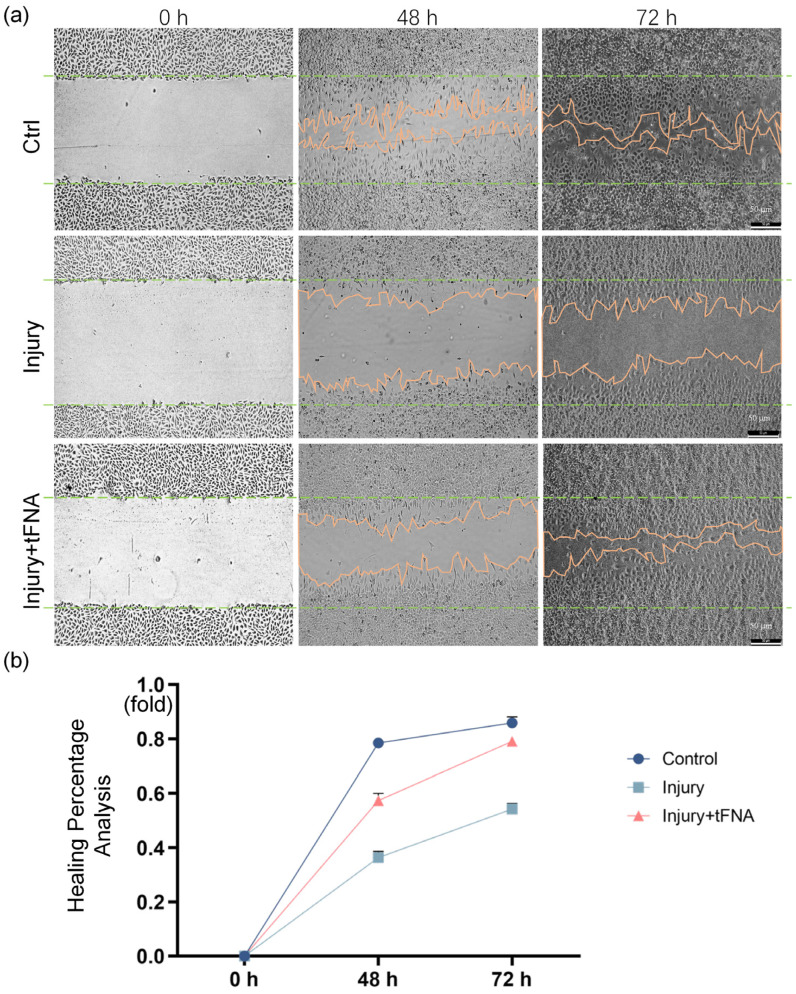
Wound healing closure assay and semiquantitative analysis after tFNA treatment. (**a**) The migration ability of damaged cells under tFNA treatment was enhanced. (**b**) Corresponding fluorescence semiquantitative measurement. Data are presented as mean ± standard deviation (SD) (*n* = 4).

After confirmation of cell proliferation, we evaluated their migratory ability using a scratch assay (Figure 11a,b). Within 72 h after scratch modeling, the undamaged cells in the control group migrated normally and gradually to fill the scratch defects, and the healing ratio reached 78.50% and 85.89% at 48 and 72 h, respectively, whereas the cells in the injury group had a reduced migratory ability, and the healing ratio reached only 36.34% and 54.27% at 48 and 72 h, respectively. The migration ability of the damaged cells under tFNA treatment reached 1.57 times (57.37% healing percentage) that of the injury group at 48 h and 1.45 times (79% healing percentage) that of the injury group at 72 h.

By combining the results of CCK-8, flow cytometry, and partial PCR, we can see that framework nucleic acids activate the proliferative potential of damaged cells by promoting the expression of genes related to the proliferative cycle and accelerate the recovery of activity. The increase in CCND1, CCND2, and Cyc-B expression after treatment with framework nucleic acids indicates the recovery of cell proliferation activity, which is consistent with the cell cycle results measured during the flow cycle. The changes in the percentage of cells in the S phase and G2 phase of the cell cycle indicated that damaged cell activity was restored to some extent under framework nucleic acid treatment. In addition to restoring cell viability, the scratch assay confirmed the enhancement of cell migration ability. The restoration of the proliferation and migration ability of burn-damaged cells is crucial for the early closure of burn wounds and the restoration of their morphological integrity by ensuring the re-establishment of their barrier function and laying the structural foundation for the subsequent restoration of electrolyte balance and immune function [32,33,34].

### 3.6. In Vivo Modeling to Validate the Healing Effect of Framework Nucleic Acids on Burn Wounds

**Figure 12:**
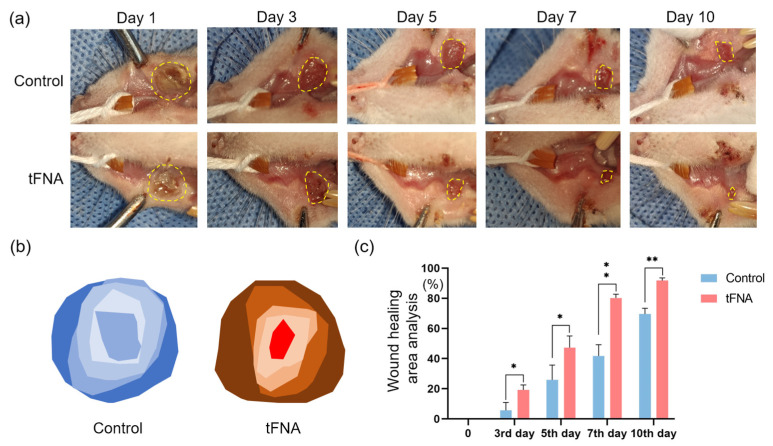
In vivo pro-healing effect of tFNA on oral mucosal burn wounds. (**a**) Photographs of full-thickness burn wounds treated with PBS and tFNA on the 1st, 5th, 7th, and 10th days after treatment. Yellow dashed circles points to the wound contour. (**b**,**c**) Calculation and comparison of ulcer-healing area of blank and tFNA groups. tFNA effectively promoted wound closure throughout the full cycle of post-injury healing. Data are presented as mean ± SD (*n* = 4). Statistical analysis: * *p* < 0.05; ** *p* < 0.01.

After completing the in vitro exploration and validation at the material–molecule–cell level, we further tested an in vivo model for the linkage and integration of the material–molecule–cell–animal system to confirm the clinical potential of the framework nucleic acids. In this study, the healing effect of tetrahedrons on oral mucosal burns was further investigated in vivo. Sodium hydroxide pellets were cut with a mold in a dry environment and placed on the mucosa for injury, after which they were administered around the injury area and photographed for documentation and measurement. As shown in the wound healing graphs (Figure 12a–c), the tFNA was effective at promoting wound closure throughout the full cycle of post-injury healing. At the time points, tFNA treatment increased the percentage of wound healing from 5.69%, 25.96%, 41.83%, and 69.59% (the injury group) to 19.22%, 47.26%, 80.21%, and 91.78% (the injury + tFNA group) at the 3rd, 5th, 7th, and 10th day, respectively. The tFNA group healed faster than the injury group over time.

**Figure 13:**
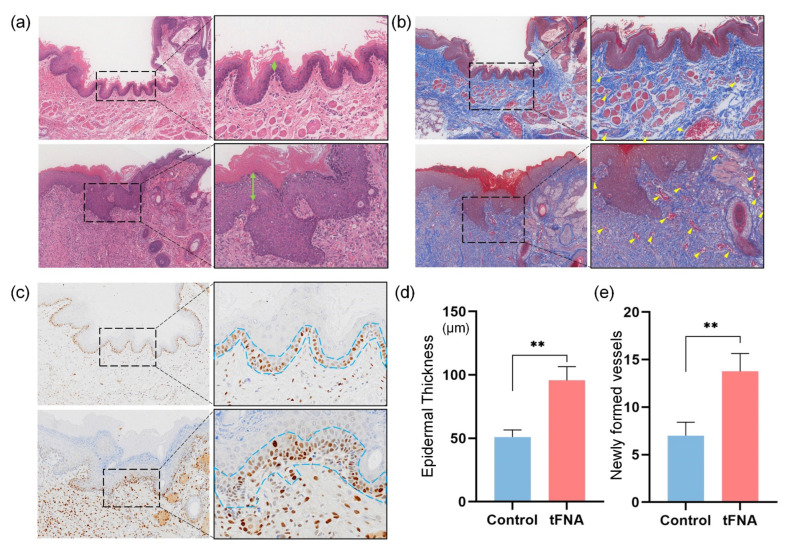
Analysis of wound healing in tissue samples. (**a**,**b**) HE and Masson staining images of buccal mucosa sections of blank and tFNA groups. Green arrows in (**a**) point to the epidermal thickness and yellow arrows in (**b**) point to vessels. (**c**) Immunohistochemical analysis of PCNA proteins from sections of blank and tFNA groups. (**d**,**e**) Statistical analysis of average epidermal thickness and micro-vessels. Data are presented as mean ± SD (*n* = 4). Statistical analysis: ** *p* < 0.01.

Subsequent HE and Masson staining (Figure 13a,b,d,e) further analyzed the healed structures. The tFNA group healed with thicker epidermal regeneration (95.69 μm in the tFNA group and 51.11 μm in the injury group) and more blood vessels than the injury group (17.75 in the tFNA group and 7 in the injury group). Additionally, the amount of orderly and arranged collagen fibers resulted in higher-quality healing.

The nano-properties of framework nucleic acids and the tetrahedral shape of the sharp-angle structure have laid the foundation for its good mucous membrane penetration, and its deeper penetration ability can regulate cell proliferation, migration, and oxidation–antioxidant balance at multiple levels faster, more extensively, and more synchronously, laying the foundation for faster and more orderly wound healing. This is corroborated by the more rapid healing of burn wounds under the action of framework nucleic acids found in subsequent results. The staining (Figure 13c) of PCNA proteins (cell proliferation-related proteins) was refined to verify the cell cycle regulation by framework nucleic acids in the in vitro experiments, which promoted the entry of the cells localized in the wounds into the proliferation cycle.

## 4. Conclusions

**Figure 14:**
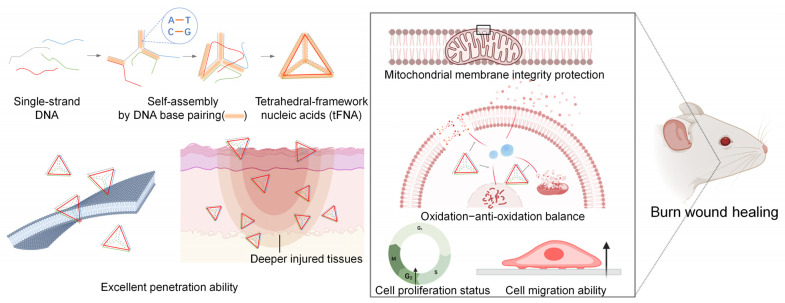
Schematic illustration showing high penetration and accelerated wound healing effect of tetrahedral-framework nucleic acid nanozymes. The construction of tFNA nanozymes penetrates deep injured tissues, protects mitochondrial structural and functional integrity, and down-regulates reactive oxygen species production during repair processes.

In summary (Figure 14), we demonstrated the accelerated wound healing of tetrahedral-framework nucleic acid (tFNA) nanozymes with high penetration and antioxidant capacity. The designed tFNA nanozymes were able to penetrate the skin as deep as 450 μm within 24 h. This remarkable penetration was beneficial to protect the mitochondrial structural and functional integrity and down-regulate the reactive oxygen species production to repair oxidative imbalances through ERK1/2-Nrf2-HO-1 during repair processes. In vitro and in vivo results indicated that tFNA nanozymes promoted the entry of postburn cells into the proliferative cycle and accelerated their migration capacity, eventually busting the early closure of wounds. This study provides a promising therapeutic strategy for burn wound healing in patients with oxidative environment disorders in the deep layers.

## Figures and Tables

**Table 1:** Sequences for synthesis preparation.

Single Strands	5′-to-3′ Base Sequence
ssDNA 1	ATTTATCACCCGCCATAGTAGACGTATCACCA GGCAGTTGAGACGAACATTCCTAAGTCTGAA
ssDNA 2	ACATGCGAGGGTCCAATACCGACGATTACAG CTTGCTACACGATTCAGACTTAGGAATGTTCG
ssDNA 3	ACTACTATGGCGGGTGATAAAACGTGTAGCAA GCTGTAATCGACGGGAAGAGCATGCCCATCC
ssDNA 4	ACGGTATTGGACCCTCGCATGACTCAACTGCC TGGTGATACGAGGATGGGCATGCTCTTCCCG

**Table 2:** Primers sequences for q-PCR.

Genes	5′-to-3′ Base Sequence
*NQO1*	F: TCAGAGAAGACATCATTCAACTACGC R: AAGTTCATAGCATAGAGGTCAGATTCG
*GSH-Px*	F: AGTCCACCGTGTATGCCTTCTC R: CGGATCGTGGTGCCTCAGAG
*SOD*	F: GTTACAACTCAGGTCGCTCTTCAG R: TGATAGCCTCCAGCAACTCTCC
*CCND1*	F: TCTCCGCCCGCCTCTCC R: CCAGCCTCTTCCTCCACTTCC
*CCND2*	F: ATTGTATTTGATGGCATCGCTC R: ATTCATTCCCTGCAAAGAACAC
*Cyc-B*	F: CTTGAAAGCGAGGAAGAAGGAGTG R: CAGAGCAGAGCATCAGAGAAAGC

## Data Availability

The original contributions presented in the study are included in this article; further inquiries can be directed to the corresponding author.

## References

[1] Jeschke M.G., van Baar M.E., Choudhry M.A., Chung K.K., Gibran N.S., Logsetty S. (2020). Burn injury. Nat. Rev. Dis. Primers.

[2] Finnerty C.C., Jeschke M.G., Branski L.K., Barret J.P., Dziewulski P., Herndon D.N. (2016). Hypertrophic scarring: The greatest unmet challenge after burn injury. Lancet.

[3] Uberoi A., McCready-Vangi A., Grice E.A. (2024). The wound microbiota: Microbial mechanisms of impaired wound healing and infection. Nat. Rev. Microbiol..

[4] Zöller K., To D., Bernkop-Schnürch A. (2024). Biomedical applications of functional hydrogels: Innovative developments, relevant clinical trials and advanced products. Biomaterials.

[5] Liang Y., He J., Guo B. (2021). Functional Hydrogels as Wound Dressing to Enhance Wound Healing. ACS Nano.

[6] Beach M.A., Nayanathara U., Gao Y., Zhang C., Xiong Y., Wang Y., Such G.K. (2024). Polymeric Nanoparticles for Drug Delivery. Chem. Rev..

[7] Yeo D.C., Wiraja C., Paller A.S., Mirkin C.A., Xu C. (2018). Abnormal scar identification with spherical-nucleic-acid technology. Nat. Biomed. Eng..

[8] Wiraja C., Zhu Y., Lio D.C.S., Yeo D.C., Xie M., Fang W., Li Q., Zheng M., Van Steensel M., Wang L. (2019). Framework nucleic acids as programmable carrier for transdermal drug delivery. Nat. Commun..

[9] Hu Q., Li H., Wang L., Gu H., Fan C. (2019). DNA Nanotechnology-Enabled Drug Delivery Systems. Chem. Rev..

[10] Li S., Liu Y., Zhang T., Lin S., Shi S., He J., Xie Y., Cai X., Tian T., Lin Y. (2022). A Tetrahedral Framework DNA-Based Bioswitchable miRNA Inhibitor Delivery System: Application to Skin Anti-Aging. Adv. Mater..

[11] Tian T., Zhang T., Shi S., Gao Y., Cai X., Lin Y. (2023). A dynamic DNA tetrahedron framework for active targeting. Nat. Protoc..

[12] Zhang T., Tian T., Zhou R., Li S., Ma W., Zhang Y., Liu N., Shi S., Li Q., Xie X. (2020). Design, fabrication and applications of tetrahedral DNA nanostructure-based multifunctional complexes in drug delivery and biomedical treatment. Nat. Protoc..

[13] Li H., Li B., Lv D., Li W., Lu Y., Luo G. (2023). Biomaterials releasing drug responsively to promote wound healing via regulation of pathological microenvironment. Adv. Drug. Deliv. Rev..

[14] Liu J., Han X., Zhang T., Tian K., Li Z., Luo F. (2023). Reactive oxygen species (ROS) scavenging biomaterials for anti-inflammatory diseases: From mechanism to therapy. J. Hematol. Oncol..

[15] Las Heras K., Garcia-Orue I., Rancan F., Igartua M., Santos-Vizcaino E., Hernandez R.M. (2024). Modulating the immune system towards a functional chronic wound healing: A biomaterials and Nanomedicine perspective. Adv. Drug. Deliv. Rev..

[16] Sanjarnia P., Picchio M.L., Polegre Solis A.N., Schuhladen K., Fliss P.M., Politakos N., Metterhausen L., Calderón M., Osorio-Blanco E.R. (2024). Bringing innovative wound care polymer materials to the market: Challenges, developments, and new trends. Adv. Drug. Deliv. Rev..

[17] Stern M., McNew J.A. (2021). A transition to degeneration triggered by oxidative stress in degenerative disorders. Mol. Psychiatry.

[18] Xu H., Guo Y., Liu X.J., Liu Y., Yin S., Bao Q.Y., Peng R., Tian W.B., Xia Y.Y., Gao L. (2024). Idebenone Antagonizes P53-Mediated Neuronal Oxidative Stress Injury by Regulating CD38-SIRT3 Protein Level. Neurochem. Res..

[19] Duan Y., Deng M., Liu B., Meng X., Liao J., Qiu Y., Wu Z., Lin J., Dong Y., Duan Y. (2024). Mitochondria targeted drug delivery system overcoming drug resistance in intrahepatic cholangiocarcinoma by reprogramming lipid metabolism. Biomaterials.

[20] Xiang Q., Yi X., Zhu X.H., Wei X., Jiang D.S. (2024). Regulated cell death in myocardial ischemia-reperfusion injury. Trends Endocrinol. Metab..

[21] Foo J., Bellot G., Pervaiz S., Alonso S. (2022). Mitochondria-mediated oxidative stress during viral infection. Trends Microbiol..

[22] El Kebbaj R., Bouchab H., Tahri-Joutey M., Rabbaa S., Limami Y., Nasser B., Egbujor M.C., Tucci P., Andreoletti P., Saso L. (2024). The Potential Role of Major Argan Oil Compounds as Nrf2 Regulators and Their Antioxidant Effects. Antioxidants.

[23] Cheng J., Xu J., Gu Y., Wang Y., Wang J., Sun F. (2024). Melatonin ameliorates 10-hydroxycamptothecin-induced oxidative stress and apoptosis via autophagy-regulated p62/Keap1/Nrf2 pathway in mouse testicular cells. J. Pineal. Res..

[24] Geertsema S., Bourgonje A.R., Fagundes R.R., Gacesa R., Weersma R.K., van Goor H., Mann G.E., Dijkstra G., Faber K.N. (2023). The NRF2/Keap1 pathway as a therapeutic target in inflammatory bowel disease. Trends Mol. Med..

[25] Zheng F., Ye C., Lei J.Z., Ge R., Li N., Bo J.H., Chen A.D., Zhang F., Zhou H., Wang J.J. (2024). Intervention of Asprosin Attenuates Oxidative Stress and Neointima Formation in Vascular Injury. Antioxid. Redox. Signal..

[26] Yuchen Z., Du M.R., Zhang Q.Y., Yang S.Y., Chen J.Q., Dan C.M., Lian L.D., Wang J. (2024). Armillariella tabescens-derived polysaccharides alleviated Ɒ-Gal-induced neuroinflammation and cognitive injury through enterocerebral axis and activation of keap-1/Nrf2 pathway. Int. J. Biol. Macromol..

[27] Acevedo S., Covarrubias A.A., Haeger P., Pancetti F., Tala F., de la Fuente-Ortega E. (2024). Alginate Oligosaccharides Protect Gastric Epithelial Cells Against Oxidative Stress Damage Through Induction of the Nrf2 Pathway. Antioxidants.

[28] Qi J., Zhou S., Wang G., Hua R., Wang X., He J., Wang Z., Zhu Y., Luo J., Shi W. (2024). The Antioxidant Dendrobium officinale Polysaccharide Modulates Host Metabolism and Gut Microbiota to Alleviate High-Fat Diet-Induced Atherosclerosis in ApoE(−/−) Mice. Antioxidants.

[29] He Q., Feng W., Chen X., Xu Y., Zhou J., Li J., Xu P., Tang Y. (2024). H_2_O_2_-Induced Oxidative Stress Responses in Eriocheir sinensis: Antioxidant Defense and Immune Gene Expression Dynamics. Antioxidants.

[30] Miao H., Tang X., Cui Y., Shi J., Xiong X., Wang C., Zhang Y. (2024). Obeticholic Acid Inhibit Mitochondria Dysfunction Via Regulating ERK1/2-DRP Pathway to Exert Protective Effect on Lipopolysaccharide-Induced Myocardial Injury. Adv. Biol..

[31] Liu N., Liang Y., Wei T., Huang X., Zhang T., Tang M. (2024). ROS-mediated NRF2/p-ERK1/2 signaling-involved mitophagy contributes to macrophages activation induced by CdTe quantum dots. Toxicology.

[32] Peña O.A., Martin P. (2024). Cellular and molecular mechanisms of skin wound healing. Nat. Rev. Mol. Cell. Biol..

[33] Kanaujiya S., Arya D.K., Pandey P., Singh S., Pandey G., Anjum S., Anjum M.M., Ali D., Alarifi S., Mr V. (2024). Resveratrol-Ampicillin Dual-Drug Loaded Polyvinylpyrrolidone/Polyvinyl Alcohol Biomimic Electrospun Nanofiber Enriched with Collagen for Efficient Burn Wound Repair. Int. J. Nanomed..

[34] Brückner D.B., Broedersz C.P. (2024). Learning dynamical models of single and collective cell migration: A review. Rep. Prog. Phys..

